# Population genomics of invasive rodents on islands: Genetic consequences of colonization and prospects for localized synthetic gene drive

**DOI:** 10.1111/eva.13210

**Published:** 2021-03-10

**Authors:** Kevin P. Oh, Aaron B. Shiels, Laura Shiels, Dimitri V. Blondel, Karl J. Campbell, J. Royden Saah, Alun L. Lloyd, Paul Q. Thomas, Fred Gould, Zaid Abdo, John R. Godwin, Antoinette J. Piaggio

**Affiliations:** ^1^ National Wildlife Research Center USDA APHIS Wildlife Services Fort Collins Colorado USA; ^2^ Department of Microbiology, Immunology and Pathology Colorado State University Fort Collins Colorado USA; ^3^ Department of Biological Sciences North Carolina State University Raleigh North Carolina USA; ^4^ Island Conservation Puerto Ayora Ecuador; ^5^ School of Agriculture and Food Sciences The University of Queensland Gatton Queensland Australia; ^6^ Genetic Engineering and Society Center North Carolina State University Raleigh North Carolina USA; ^7^ Biomathematics Graduate Program and Department of Mathematics North Carolina State University Raleigh North Carolina USA; ^8^ The Robinson Research Institute and School of Medicine The University of Adelaide Adelaide South Australia Australia; ^9^ Department of Entomology and Plant Pathology North Carolina State University Raleigh North Carolina USA

**Keywords:** Cas9, CRISPR, genetic biocontrol, genome editing, *Mus musculus*, synthetic biology

## Abstract

Introduced rodent populations pose significant threats worldwide, with particularly severe impacts on islands. Advancements in genome editing have motivated interest in synthetic gene drives that could potentially provide efficient and localized suppression of invasive rodent populations. Application of such technologies will require rigorous population genomic surveys to evaluate population connectivity, taxonomic identification, and to inform design of gene drive localization mechanisms. One proposed approach leverages the predicted shifts in genetic variation that accompany island colonization, wherein founder effects, genetic drift, and island‐specific selection are expected to result in locally fixed alleles (LFA) that are variable in neighboring nontarget populations. Engineering of guide RNAs that target LFA may thus yield gene drives that spread within invasive island populations, but would have limited impacts on nontarget populations in the event of an escape. Here we used pooled whole‐genome sequencing of invasive mouse (*Mus musculus*) populations on four islands along with paired putative source populations to test genetic predictions of island colonization and characterize locally fixed Cas9 genomic targets. Patterns of variation across the genome reflected marked reductions in allelic diversity in island populations and moderate to high degrees of differentiation from nearby source populations despite relatively recent colonization. Locally fixed Cas9 sites in female fertility genes were observed in all island populations, including a small number with multiplexing potential. In practice, rigorous sampling of presumptive LFA will be essential to fully assess risk of resistance alleles. These results should serve to guide development of improved, spatially limited gene drive design in future applications.

## 1 INTRODUCTION

Invasive rodent populations occupy more than 80% of islands worldwide where they commonly pose significant threats to endemic biodiversity, as well as agricultural production and human health (Howald et al., 2007; Jones et al., 2016; Meerburg et al., 2009). Management efforts to date have relied heavily on chemical rodenticides, which can often be prohibitively expensive or logistically infeasible for many island applications, and also incur substantial costs in terms of environmental burden and off‐target species mortality (Nakayama et al., 2019). These shortcomings, along with the advent of precision genome editing afforded by CRISPR‐Cas technologies, have motivated interest in the development of synthetic gene drives for rodent population suppression (Campbell et al., 2015; Godwin et al., 2019; Gould, 2008; Piaggio et al., 2017; Rode et al., 2019). Unlike most toxicant‐based management methods, gene drives are strictly transmitted through inheritance and thus species specificity is largely ensured by assortative mating among conspecifics. Such specificity is particularly important for islands with human habitation or species of conservation concern where the use rodenticides is often restricted due to impacts on nontarget species. Moreover, the self‐replicating nature of homing endonuclease gene drive systems, wherein target sequences are cut and the gene drive elements copied to the homologous chromosome via homology directed repair (HDR or “homing”), is an attractive feature for eradication efforts on remote or difficult to access islands where repeated treatments can be impractical (Leitschuh et al., 2018).

While a variety of gene drive designs have been proposed, the most basic strategy for suppression of wild populations involves targeting a female haplosufficient fertility or viability gene (Burt, 2003; Hammond et al., 2016; Prowse et al., 2017), wherein insertion of the gene drive construct creates a null allele. In such an approach, homing is typically confined to the germline, resulting in gene drive carriers that are somatic heterozygotes and thus viable and able to transmit the gene drive at “super‐Mendelian” proportions through sexual reproduction (Kyrou et al., 2018). Targeting fertility genes that only affect females is expected to facilitate faster spread of the gene drive via carrier reproductive males (Deredec et al., 2008), especially in species such as rats and mice where multiple paternity is common. As the gene drive spreads, population suppression is achieved through the inviability or infertility of increasingly frequent homozygous individuals.

Given the ability of synthetic gene drives to propagate rapidly within and among populations, the development of safeguards to limit spread to nontarget populations is a key technological challenge (Dhole et al., 2018; Noble et al., 2018), as the ecological impacts of uncontrolled spread outside of the treatment area may present an unacceptable risk (Gould, 2008). Several molecular strategies have been proposed to limit gene drive spread including physical separation of gene drive components (“split drive,” DiCarlo et al. 2015) or mechanisms such as toxin‐antidote designs (Champer et al., 2020a) or engineered underdominance (Dhole et al., 2019; Reeves et al., 2014) that permit drive spread only above a certain population frequency threshold (Leftwich et al., 2018).

Another proposed approach capitalizes on the precise genome editing afforded by CRISPR‐Cas systems to target polymorphic sequences that are fixed (allele frequency = 1.0) in the population of interest (i.e., locally fixed alleles, LFA), but absent (or at lower frequency) in nontarget populations (Sudweeks et al., 2019; Teem et al., 2020). Evidence suggests that a single nucleotide change in either the protospacer adjacent motif (PAM) (Hsu et al., 2013) or anywhere within the “core” (four nucleotides at position +4 to +7 upstream of the PAM, Zheng et al., 2017) of a Cas9 guide RNA (gRNA) target site can be sufficient to preclude endonuclease binding. Thus, population specificity might be accomplished through designing gRNA that bind sequences that are present in the target populations but absent in nontarget populations. Recent modeling by Sudweeks et al. (2019) demonstrates that the Locally fixed alleles approach can effectively achieve localized population suppression under a variety of conditions. Moreover, this work suggests that escape and interbreeding of drive‐bearing individuals out of the treatment area is likely to result in only transient suppression of nontarget populations, even in “worst case scenarios” when a susceptible (i.e., target) allele is present at a high frequency (0.95) in the nontarget population. This phenomenon is explained by the presence of “resistance” alleles, which in this case are naturally occurring genetic polymorphisms in the target sequence that effectively inhibit cleavage. These resistance alleles are expected to be rapidly driven to high frequencies as a result of selection against drive‐bearing individuals. This finding also emphasizes the critical importance of thorough genetic study of the target population prior to gRNA design to identify sequences that are locally invariant, as even a low level of polymorphism would reduce efficacy of gene drive mediated population suppression.

In addition to resistance from standing genetic variation, recent studies (Champer et al., 2017; Unckless et al., 2017) have demonstrated that resistance will also inevitably arise within populations from de novo mutations in the target site, or by the gene drive itself as a consequence of errors in the cleavage repair process (e.g., nonhomologous end joining, NHEJ). Gene drive designs that target coding sequences (CDS) of fertility or viability genes may afford some protection from resistance if NHEJ creates loss‐of‐function mutations, which will be selected against in the homozygous state or when inherited alongside the gene drive construct. Another proposed solution to the evolution of resistance is the design of drive systems with multiplexed gRNA (Champer et al., 2018), that is, multiple gRNA that each target closely spaced (ideally <500 bp) genomic regions, thereby decreasing the likelihood of resistance gene drive disruption from resistance arising at any single site (Oberhofer et al., 2018). Indeed, evidence from *in silico* modeling suggests that multiplexed gRNA is likely to be necessary for successful population suppression, even under low levels of NHEJ (Champer, Oh, et al., 2020; Prowse et al., 2017). Experimental work in insects suggests, however, that the benefits of additional gRNAs may be limited, and there is likely an optimal number (in this case, between two and eight) that balances tolerance to resistance with overall drive conversion efficiency (Champer, Oh, et al., 2020).

The feasibility of the LFA strategy for gene drive localization in the context of vertebrate pest management will depend critically on several aspects of population genetic structure and ecological setting. As gene drive efficacy will be diminished by ongoing immigration of resistant individuals, relatively isolated populations with low levels of gene flow to nontarget populations, such as remote oceanic islands, would provide ideal settings. Introduced island populations, often characterized by small numbers of founding individuals and susceptibility to genetic drift, are expected to harbor reduced allelic diversity (Frankham, 1997), thereby providing a relatively high frequency of LFA targets. Moreover, previous theoretical and empirical work suggests that island habitation might impose novel selection for island‐adapted phenotypes in newly introduced populations (i.e., “island syndrome,” Adler & Levins, 1994; Foster, 1964), which in some cases could involve selective sweeps that lead to fixation of alleles which could in turn serve as LFA targets. However, these assumptions require empirical validation and population genetic characterization of potential targeted island populations, as well as nontarget populations along hypothetical escape pathways.

Here we perform population genomic analyses of introduced house mice (*Mus musculus*) on islands to understand patterns of genomic variation associated with colonization, and to test key assumptions underlying the LFA gene drive strategy. House mice are comprised of three primary subspecies: the western house mouse (*M*.* m*.* domesticus*), eastern house mouse (*M*.* m*.* musculus*), and the southeast Asian house mouse (*M*.* m*.* castaneus*), with varying levels of gene flow among lineages at contact zones (Bonhomme et al., 2007). House mice are among the most broadly distributed invasive vertebrate species, primarily dispersed through commensal relationships with humans (Boursot et al., 1993). While perhaps less conspicuous a threat than other rodent species (e.g., *Rattus* spp.), a recent survey identified at least 35 islands with endangered or critically endangered species where house mice were the only invasive rodent present (Threatened Island Biodiversity Database, http://tib.islandconservation.org/). At present, control of invasive mice on islands relies almost exclusively on anticoagulant rodenticides, which can often be effective, but also face limitations due to lack of species specificity, high costs of application, and persistence in the environment (Godwin et al., 2019). Interest in the application of gene drive for control of invasive mouse populations on islands has been motivated not only by their ubiquity and severity of ecosystem impacts (Angel et al., 2009), but also by the availability of genomic resources due to the status of *M*.* m*.* domesticus* as a model research organism. Recent CRISPR‐Cas9 experiments in mice have demonstrated success in generating double‐stranded breaks at target sequences (Pfitzner et al., 2020), as well as homology directed repair to increase rates of inheritance (Grunwald et al., 2019), though not yet to a degree of efficiency necessary for biocontrol applications. Thus, while substantial technical challenges remain, evidence suggests that mice may likely be the first vertebrate species for which a working gene drive system is achieved, which will also serve as an important model for gene drive development in other rodents.

Unlike many population genetic applications where parameters can be reliably estimated by querying a relatively small number of molecular markers, designing targeted gene drives based on scans for LFA relies on the ability to query the entire genome, which can be prohibitively costly in terms of sequencing and library preparation. Thus, we utilize a pooled sequencing approach (“pool‐seq,” Schlötterer et al. 2014), which has been demonstrated to provide greater precision in population allele frequency estimates compared to individual‐based sequencing at equivalent effort over a range of experimental conditions (Rode et al., 2018). Pooled sequencing is applied here to evaluate the population genetic consequences of island colonization with respect to the frequency of LFA targets.

## 2 METHODS AND MATERIALS

### 2.1 Sample collection and DNA extraction

**FIGURE 1:**
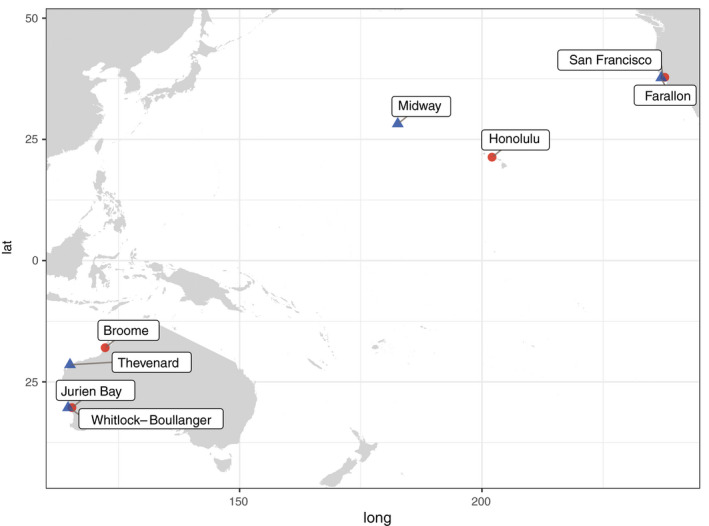
Map of island (blue triangles) and “source” (red circles) mouse study populations

All aspects of the study were approved by the Institutional Animal Care and Use Committees of the USDA National Wildlife Research Center and North Carolina State University and were performed in accordance with institutional policy and National Institutes of Health guidelines governing the humane treatment of vertebrate animals. Invasive mouse populations were sampled on four islands: Southeast Farallon Island (39 ha), Sand Island (510 ha), Thevenard Island (550 ha), and Whitlock–Boullanger Islands (37 ha together, joined at low tides) (Figure 1). These populations were selected based on criteria established from an exercise aimed at identifying suitable islands for a hypothetical future gene drive deployment (for details of island selection criteria, see Campbell et al. 2019). For each island population, we attempted to sample a paired “source” location that represented a nontarget population to which a gene drive‐bearing island mouse might likely escape or that may share similar genetic profiles. These selections were based on expert opinion and the assumption that movement of mice was likely to be human‐mediated and were thus not necessarily the nearest in terms of geographic proximity (approximate interpopulation distances estimated using https://www.nhc.noaa.gov/gccalc.shtml are provided in Table S1). In the case of Midway Atoll, for example, where nearly all anthropogenic traffic to and from the island is via aircraft, the Honolulu Airport on Oahu, Hawai'i, was selected as the source population. All source locations are characterized by an established human presence and are assumed to represent relatively large, genetically diverse mouse populations. We note that, for Thevenard Island in Western Australia, we were unable to acquire adequate numbers of samples at the closest mainland population (Onslow), and thus relied upon mice collected from Broome, a larger coastal city approximately 850 km to the north.

Mouse tissues for this study (summarized in Table S1) were obtained through a combination of live and kill trapping in the course of pest control operations, as well as samples obtained under a tissue grant from the Museum of Vertebrate Zoology, University of California, Berkeley (Table S2). Genomic DNA was isolated using column‐based methods (DNeasy Blood and Tissue Kits; Qiagen, Inc.) following the manufacturer's recommended protocol. DNA purity was assessed by inspecting the A260/A280 ratio for each sample on a NanoDrop ND‐1000 spectrophotometer (Thermo Fisher Scientific Inc.).

### 2.2 Pooled whole‐genome resequencing

Individual genomic DNA samples were combined into population‐specific pools for whole‐genome resequencing. Accurate estimation of population allele frequencies from pool‐seq experiments is strongly dependent on equal representation of all individuals in the sequencing library (Rode et al., 2018). Thus, to minimize variation among individual representation within pools, DNA was quantified in triplicate using fluorometric assays (Quant‐iT PicoGreen dsDNA Assay; Thermo Fisher Scientific, Inc.) prior to pooling. Whole‐genome shotgun libraries were prepared separately for each pool (TruSeq DNA PCR‐free Library Prep Kits; Illumina, Inc.) and then submitted to MedGenome Labs (MedGenome, Inc.) for paired‐end 150 bp sequencing on a HiSeq X instrument (Illumina, Inc), one library per lane, for a targeted 40× mean sequencing depth per pool (i.e., 1× per individual in 40‐sample pools).

### 2.3 Bioinformatic processing and population genomic analyses

Preprocessing of raw sequence data was carried out in GATK4 following the “best practices” workflow (Van der Auwera et al., 2013). Briefly, sequencing adapters were marked using the MarkIlluminaAdapters tool in Picard v2.20.2 (http://broadinstitute.github.io/picard/), followed by mapping to the GRCm38/mm10 mouse reference assembly (https://www.ncbi.nlm.nih.gov/grc) using bwa v0.7.12 (Li, 2013). To account for misalignment caused by indels, mapped reads were subject to local realignment using the IndelRealigner tool in GATK4. Final cleaned aligned sequence files were generated using the MergeBamAlignment tool.

Genetic diversity within each population was estimated as expected SNP heterozygosity (SNP‐*H*_e_) across all autosomal biallelic sites following the method proposed by Fischer et al. (2017) which assumes Hardy–Weinberg equilibrium within populations. Additionally, mean Watterson's *θ* (Watterson, 1975) and Tajima's *D* (Tajima, 1989) were calculated across all autosomal exonic regions using the Variance‐at‐position.pl Perl script in PoPoolation (Kofler, Orozco‐terWengel, et al., 2011), along with the curated NCBI RefSeq mouse genome annotation file downloaded from the UCSC Table Browser (Karolchik et al., 2004). Tajima's *D* is a statistic primarily utilized to test for evidence of non‐neutral evolution, but may be also affected by demographic processes including population bottlenecks or expansion (Tajima, 1989). Sites were filtered for a minimum base quality of 20 and a coverage range from 20 to 1000×. Only exons with at least 60% of bases falling within the coverage range across all populations were included. Estimates from exonic regions are considered to be conservative estimates of population diversity as most genes are expected to be subject to stabilizing selection.

To evaluate overall patterns of genetic divergence across all populations in the dataset, we performed principal component analysis on autosomal SNPs using the pcadapt R package (Luu et al., 2017). Genome‐wide allelic differentiation (*F*_ST_), which ranges from 0 (complete panmixis) to 1 (no shared genetic diversity), between paired population samples was estimated in R using the ANOVA method in the poolfstat package (Hivert et al., 2018) after creating mpileup files from the mapped reads using SAMtools v1.9 (Li, 2011) and subsequent conversion to “synchronized” format via the mpilup2sync Java utility in Popoolation2 (Kofler et al., 2011).

### 2.4 Subspecies admixture analysis

House mouse subspecies are known to interbreed at varying degrees (Bonhomme et al., 2007), and the presence of admixed individuals on islands could have implications for design of gene drive, as well as illuminating pathways of invasion. To test for genomic admixture in each population sequencing pool, we used the maximum likelihood approach implemented in iAdmix (Bansal & Libiger, 2015) along with species‐specific SNP allele frequency datasets derived from whole‐genome datasets described in Harr et al. (2016) consisting of 24 individual *M*.* m*.* domesticus* (hereafter Mmd) samples from three populations (Germany, France, Iran), 22 *M*.* m*.* musculus* (Mmm) samples from three populations (Afghanistan, Czech Republic, Kazakhstan), and 10 *M*.* m*.* castaneus* (Mmc) samples from India (reanalyzed from Halligan et al. 2010). Briefly, variant call format (VCF) files for these samples (downloaded from http://wwwuser.gwdg.de/~evolbio/evolgen/wildmouse/) were filtered for autosomal biallelic SNPs that had less than 10% missing genotypes and were present in all three species datasets, resulting in a total of 4,527,839 loci. Allele frequencies were estimated using VCFtools v0.1.16 (Danecek et al., 2011), and binary aligned sequence files for each population pool were supplied to iAdmix to estimate admixture coefficients.

### 2.5 Inferred selective sweeps

To test for evidence of selective sweeps, we applied the hidden Markov model (HMM) implemented in the Python program Pool‐hmm (Boitard et al., 2013) to each population pool‐seq dataset. Genome‐wide folded allele frequency spectra (AFS) were estimated directly from the data and subsequently supplied to the HMM to detect selection at each genomic position. The algorithm employed in this approach identifies the sequence of hidden states (“neutral,” “intermediate,” or “selection”) which maximizes the likelihood of the HMM (Boitard et al., 2012). Following the software authors’ guidelines, minimum coverage (‐c option) was set at 10, minimum base quality (‐*q*) was set at 20, the proportion of sites used to estimate the AFS (‐*r*) was set to 0.0005, the per‐site transition probability (‐*k*) was set to 1e‐10, and the starting value for AFS estimation (under constant population size and scaled mutation rate, ‐*t*) was set at 0.0018. Only regions supported by high posterior probabilities (>0.9999 for the hidden state “selection”) were retained. To further characterize the role of selection in shaping island population genetic variation, we used BEDtools v2.28.0 (Quinlan & Hall, 2010) to identify selective sweeps that were common across island populations. We then performed gene ontology enrichment analysis on this gene list using DAVID v6.8 (Huang et al., 2007) to test for the presence of enriched functional biological themes.

### 2.6 Locally fixed alleles

For the purposes of this study, we consider a standard Cas9 homing gene drive design that would target a haplosufficient female fertility gene. To identify suitable LFA, we analyzed pool‐seq data using LoFreq (Wilm et al., 2012). Compared to other available pool‐seq variant callers, LoFreq employs a statistical approach that is particularly well‐suited for efficiently detecting rare variants and singletons (Huang et al., 2015); a key feature for confidently identifying LFA. Briefly, LoFreq models sequencing run‐specific base‐call quality and mapping quality to distinguish even low‐frequency true variants from errors. Each population was analyzed separately, considering sites with a minimum mapping quality (‐‐min‐mq) of 20 and a maximum sequencing depth (‐‐d) of 10,000. Subsequent processing involving bcftools v1.9 (Li, 2011), picard v2.21.9 (http://broadinstitute.github.io/picard/), and jvarkit (Lindenbaum, 2015) was then carried out to identify SNPs that either formed functional canonical *S*.* pyogenes* Cas9 PAM sites (5′‐ NGG‐3′, where “N” is any base) or occurred anywhere within the “core” of a putative gRNA target site (i.e., nucleotide position +4 to +7 upstream from a PAM, Zheng et al., 2017). Further characterization of these potential Cas9 targets was performed using BEDtools in conjunction with the mm10 genome annotation to identify only those that occurred within a protein‐CDS or 5′UTR region, where insertion of the gene drive would be expected to form a null allele (i.e., loss‐of‐function mutation) with the desired phenotypic effect. Of these remaining candidate SNPs, we identified those that occurred within genes associated with female infertility by searching the Mouse Genome Database (www.informatics.jax.org, Bult et al., 2019) for the associated mammalian phenotype term (MP:0001926), followed by manual curation to exclude any other undesirable phenotypes (e.g., male infertility, abnormal gametogenesis, abnormal meiosis). Finally, LFA were classified using three different target allele frequency cutoffs for the “source” population. We reasoned that, while LFA with low allele frequencies (≤0.15) in the “source” population would minimize the magnitude of impact on the nontarget population in the event of gene drive escape, this would come at the cost of fewer genomic targets. Targeting LFA with high source population allele frequencies (≤0.95) might afford greater flexibility in gene drive design, and the risk of higher nontarget impacts (however transient, Sudweeks et al., 2019) might be acceptable for applications on very remote islands with robust biocontainment. A third intermediate allele frequency threshold (≤0.50) was also applied as a potential option that balances the trade‐offs of the more extreme values.

To test for correlations between LFA and selective sweeps, we performed permutation tests (1000 randomizations) using the regioneR package (Gel et al., 2015) in R. Custom scripts used for identifying and characterizing locally fixed Cas targets presented below can be accessed at https://github.com/kevin‐oh/lfa. We note that the analysis pipeline utilized here is tailored to Cas9, but is amenable to PAM sites for different Cas variants.

## 3 RESULTS

### 3.1 Pooled sequencing

We sampled mice from four pairs of island and putative source populations to perform population genomic analyses and characterize LFA. Pooled whole‐genome sequencing yielded an average of 184 giga base pairs of raw sequencing per pool (Table S3). Low initial yields for the Midway and Oahu samples necessitated additional sequencing runs, resulting in higher data yield for these two populations compared to others. Mapping to the mm10 reference genome resulted in mean coverage ranging from 40.0× to 90.0×, thus achieving the minimum recommended 1× per individual genome within each pool (Buerkle & Gompert, 2013).

**FIGURE 2:**
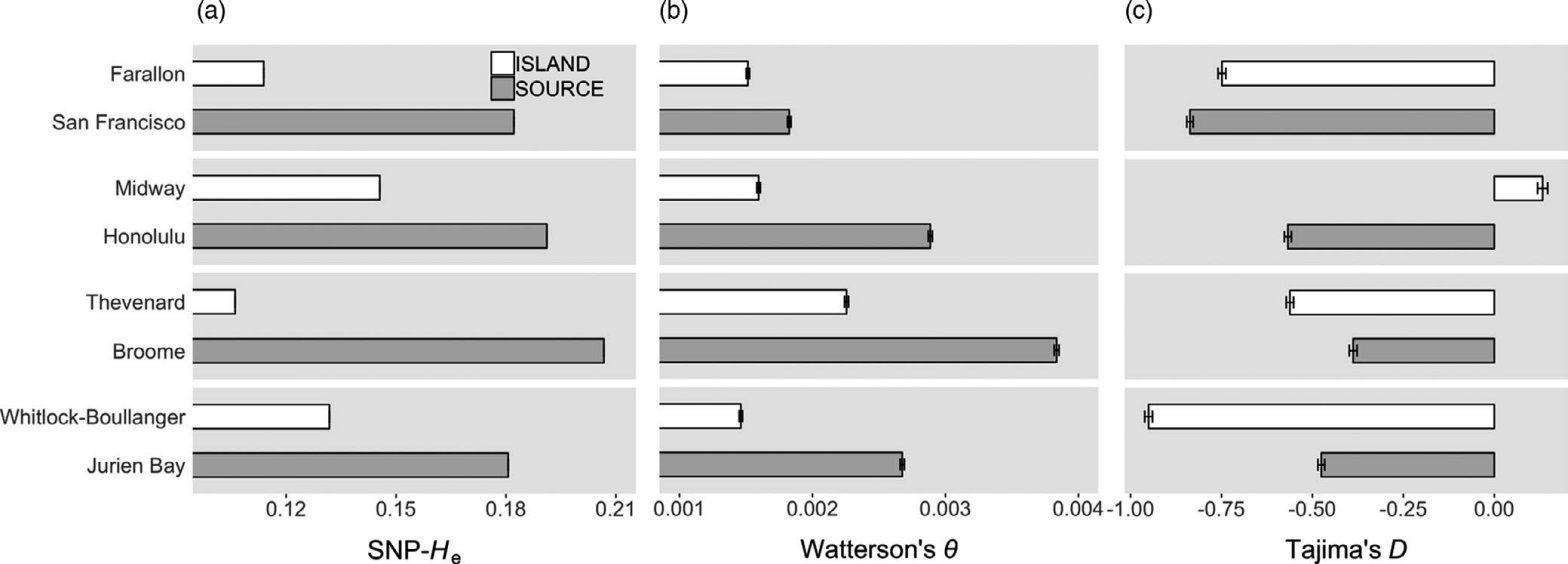
Comparison of genetic diversity in paired island (white) and putative source (gray) mouse populations: (a) mean expected heterozygosity (SNP‐*H*_e_), (b) Watterson's *θ*, and (c) Tajima's *D*. SNP‐*H*_e_ estimated across all autosomal biallelic SNPs, and *θ* and *D* calculated from the mean of autosomal exonic regions only. Error bars are standard errors of the mean (not visible in (a)). Note that in subsequent analysis of LFA, Jurien Bay is used as a proxy source population for Thevenard Island (Results)

Genome‐wide expected heterozygosity was consistently lower in island populations relative to paired source populations (Figure 2a). Likewise, estimated nucleotide diversity (Watterson's *θ*) based on 32,273 exons in the mm10 annotation showed the similar patterns of reduced diversity in island mice (Figure 2b). In tests for evidence of non‐neutral evolution, patterns of Tajima's *D* for exonic regions (Figure 2c) were inconsistent across population pairs, with all but the Midway population exhibiting negative values. We acknowledge, however, the challenge of inferring significance for *D*, as the appropriate distribution for this statistic is not known, and none of the mean values observed in our study approached the critical values recommended for rejecting neutral evolution (Simonsen et al., 1995). More generally, Tajima's *D* has been shown to lack power in analyses of population growth in some cases (Ramos‐Onsins & Rozas, 2002).

### 3.2 Population genetic structure

**FIGURE 3:**
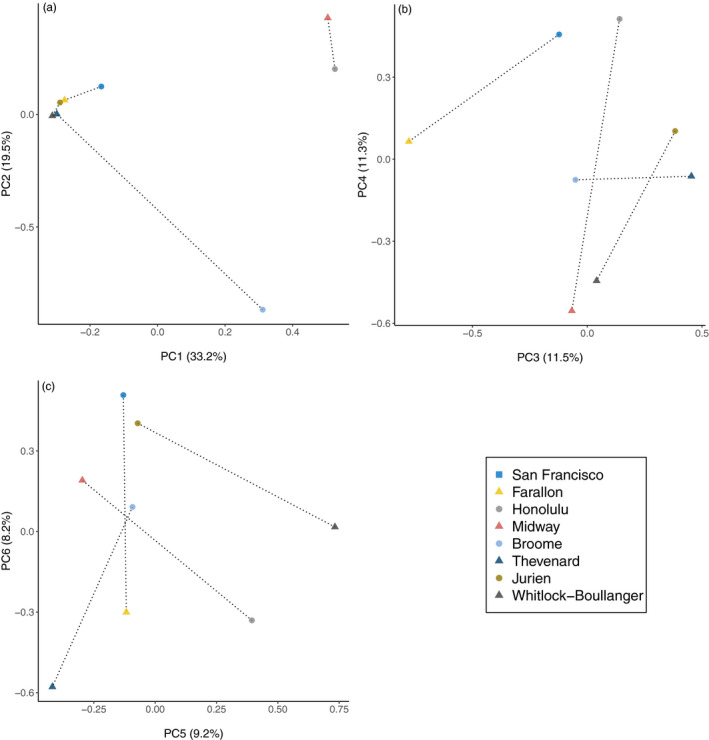
Principal component analysis of island (triangles) and putative source (circles) mouse populations at 38,412,195 autosomal SNPs. Each biplot (a through c) represents two principal components, with percent of total variation explained by each principal component in parentheses along each axis. Together, these six principal components explained the majority (92.9%) of genetic variation in the dataset. Each point represents a single pooled population sample. Broken lines connect paired populations. Note that in subsequent analysis of LFA, Jurien Bay is used as a proxy source population for Thevenard Island (Results)

PCA of autosomal SNPs across all populations (Figure 3) suggested some degree of geographic clustering of population pairs, notably Midway and Honolulu along the first two principal components (PC1 and PC2) which together accounted for a majority (52.7%) of the genetic variation in the dataset. The population from Broome also showed strong differentiation from all other populations (Figure 3a). Genetic variation along subsequent principal components, however, exhibited little discernable clustering among populations.

Pairwise *F*_ST_ from autosomal SNPs indicated modest allelic differentiation among three of the island–source pairs: Farallon Island vs San Francisco, *F*_ST_ = 0.175; Midway vs. Honolulu, *F*_ST_ = 0.151; Whitlock–Boullanger vs. Jurien, *F*_ST_ = 0.171. In contrast, differentiation between Thevenard Island and Broome mice (*F*_ST_ = 0.365) was more than twice that observed for any other population pair. Using Wright’s (1951) approximation, these values equate to migration estimates ranging from 0.44 (Midway vs. Honolulu) to 1.41 (Thevenard Island vs. Broome) migrants per generation.

### 3.3 Subspecies admixture analysis

**FIGURE 4:**
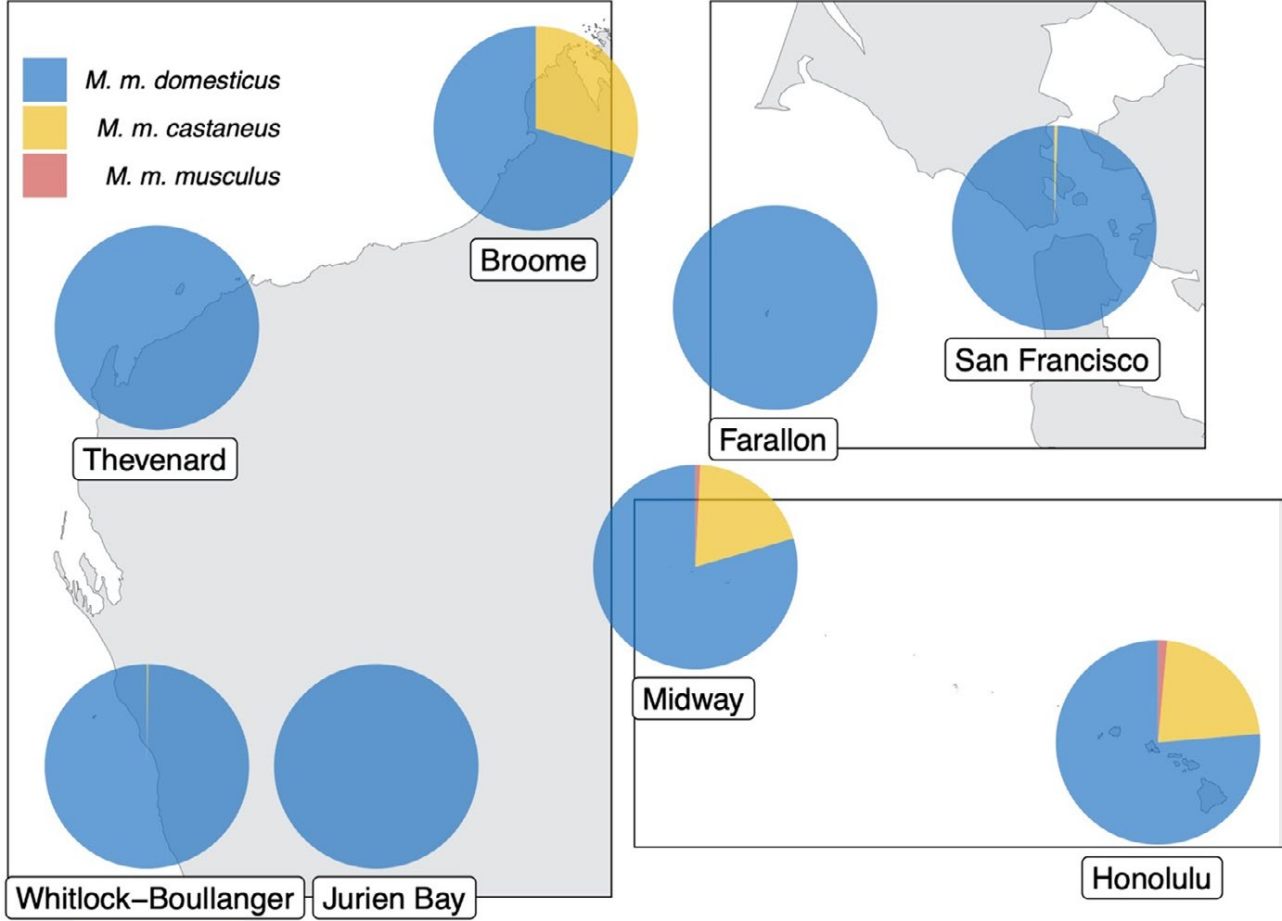
Genomic admixture of *Mus musculus* populations based on pooled whole‐genome resequencing and subspecies allele frequencies at 4,527,839 autosomal biallelic SNPs. Colors indicate admixture proportion for each subspecies. Locations on maps are approximate

Results of genomic admixture analysis (Figure 4) confirmed exclusive (or nearly exclusive, >99%) Mmd ancestry for five populations (Whitlock–Boullanger, Jurien, Thevenard, Farallon Island, and San Francisco). In contrast, both Midway and Honolulu populations exhibited Mmc ancestry in similar proportions (19.7% and 22.2%, respectively) along with 1%–2% Mmm ancestry, which supports the assumed demographic linkage between these populations. Mice from the Broome population had the highest proportion of non‐Mmd ancestry (29.6% Mmc) from any population in the dataset, and contrasts with the paired island population of Thevenard, which had exclusive Mmd ancestry.

### 3.4 Inferred selective sweeps

Analysis with Pool‐hmm identified hundreds to thousands of potential selective sweeps in each population (range 988–4756) with an average of 135.5 (SD = 59.7) sweeps inferred per autosome per population. We note that this method, which infers selective sweeps based only on deviations from genome‐wide AFS, is agnostic with respect to underlying genomic features and likely includes a proportion of false positives. Thus, because we were particularly interested in the consequences of selective sweeps on islands, we evaluated the intersection of these regions across the four island study populations, resulting in a total of 336 selective sweep regions with a combined length of 34.5 Mbp that were common to all island populations. Among these island selective sweeps, 249 overlapped with 608 protein‐coding genes in the mm10 annotation (Table S4). Gene ontology (GO) term analysis of this gene list (Table S5) revealed significant functional enrichment (*p* < 0.05, Benjamini–Hochberg adjusted) for 18 terms. Notably, four of the top ten terms were related to hormone activity, of which two involve somatotropin (growth hormone).

### 3.5 Characterization of locally fixed alleles

Results of genomic scans of pool‐seq data for LFA targets in each population pair are summarized in Table 1. Due to the relatively high genetic divergence observed between Thevenard Island and Broome mice, along with discordant subspecies ancestries, we elected to use Jurien Bay as a proxy paired source population for Thevenard in the LFA analysis as both clustered tightly in the PCA results (Figure 3), and Jurien Bay is only ~130 km further away (Euclidean distance) from Thevenard Island compared to Broome. Successive filters were applied to identify autosomal SNPs that were (1) fixed in the island population, (2) formed a *S*.* pyogenes* Cas9 PAM site or were located within the core of a potential gRNA target (nucleotide positions +4 to +7 bp upstream of existing PAM), (3) occurred within a genic CDS or 5’UTR, and (4) were associated with genes that caused female infertility (in knockout experiments). While a large number of locally fixed Cas9 target sites were observed in genic regions (mean = 8597 SNPs across islands), <2% of these were in genes associated with female infertility.

**TABLE 1:** Identification of CRISPR‐Cas9 locally fixed alleles across four island–source population pairs

SNP filter stage	Island Population			
	Farallon Isl.	Midway Atoll	Thevenard Isl.	Whitlock–Boullanger Isls.
Total autosomal SNPs	1.13e7	2.39e7	1.14e7	1.04e7
Fixed in island population	1.96e6	1.60e6	1.63e6	2.29e6
In Cas9 sites (PAM‐forming or gRNA target sequence)	683,791	759,522	750,156	1.04e6
In CDS or 5′UTR	6932	8680	8280	10,497
In female fertility gene	127	155	135	196
“Source” population allele frequency (# multiplex sets)				
AF ≤0.95	81 (8)	79 (7)	59 (7)	97 (13)
AF ≤0.50	22 (2)	8 (0)	10 (1)	18 (1)
AF ≤0.15	0	0	0	0

Note

Each row depicts number of single nucleotide polymorphisms (SNPs) after each successive filter stage (left column) and is thus a subset of the row above. LFA were identified based on three different allele frequency thresholds in the corresponding “source” population: 0.95, 0.50, and 0.15. The number of potential multiplex sets (i.e., two or more LFA SNPs occurring within a 500 bp window) are provided in parentheses. Note that for this analysis, Jurien Bay was used as a proxy “source” population for Thevenard Island (Results).

Abbreviations: 5’UTR, 5’ untranslated region; AF, SNP allele frequency; CDS, coding sequence.

Filtering of remaining SNPs for varying allele frequency cutoffs in the “source” population had substantial effects on the final numbers of LFA identified (Table 1). Applying the most stringent filter (≤0.15) excluded all loci across populations. Allowing for high allele frequencies (≤0.95) predictably resulted in the largest number of potential sites, ranging from 59 (Thevenard Island) to 97 (Whitlock–Boullanger Islands). None of these LFA identified were shared across all island populations, though 13 occurred in three populations, and 41 were observed in two. Assessment of multiplexing potential among these SNPs identified numerous sets in each population, with an overall total of one 4‐plex, six 3‐plex, and 28 2‐plex sets.

Filtering for loci with intermediate allele frequencies (≤0.50) in the “source” population resulted in counts ranging from 8 (Midway Island) to 22 (Whitlock–Boullanger Islands). Genomic locations, alleles, flanking sequences, and estimated allele frequency for each SNP are provided in Table S6. A total of five LFA were common to more than one island–source pairing, with four shared between the two Western Australian islands (Thevenard Island and Whitlock–Boullanger Islands), and the fifth LFA shared between Thevenard Island and Farallon Island. Analysis of potential for multiplexing showed limited opportunity, with zero multiplex sets evident in the Midway Island population, two 2‐plexes in the Farallon Island population, and one 2‐plex in each of the other two populations. Additionally, two of the shared LFA between Thevenard Island and Whitlock–Boullanger Islands on chromosome 18 were only separated by 552 bp (i.e., 52 bp beyond our defined multiplexing limit), indicating a potential additional multiplex option that might provide a common gene drive design for these populations (see Oberhofer et al., 2018 for detailed discussion of multiplex gRNA spacing effects). Consistent with the expected reduced diversity associated with selective sweeps, we observed significant associations between these LFA and selective sweeps within each island population: Farallon: 15 (68.2%) LFA in selective sweeps, *Z* = 3.17, *p < *0.003; Midway: 3 (37.5%), *Z* = 2.89, *p < *0.028; Thevenard: 6 (60.0%), *Z* = 2.18, *p* = 0.041; and Whitlock–Boullanger: 137 (77.8%), *Z* = 2.52, *p* = 0.010.

Detailed characterization of candidate genes that harbored LFA was carried out only for the loci with intermediate (≤0.50) “source” population allele frequencies, as this threshold arguably provides the most reasonable balance of gene drive design flexibility with minimized risk to nontarget populations. Overall, we identified 40 unique female fertility genes with Cas9 targets (either PAM‐forming SNPs or SNP‐containing gRNA core sequences) in the CDS or 5′UTR that were fixed in the island population (Table S7). Further annotation of these genes for phenotypes that might impact suitability as a gene drive target revealed a proportion (62.5%) for which there was evidence (primarily from homozygous knockout experiments) of infertility or reduced fertility in males, which may hinder the rate of spread due to the lack of reproducing carrier males (Deredec et al., 2008). Moreover, 30% of genes were associated with terms relating to abnormal gametogenesis/oogenesis or meiosis, which may also be undesirable as gene drive inheritance requires normal oogenesis in the germline of gene drive carrier females (which will be in a homozygous state due to homing). Nevertheless, this analysis highlighted three potential candidate genes with attractive characteristics: zygote arrest 1 (*Zar1*), for which we identified a 2‐plex set of LFA in the Thevenard Island population; hexokinase 1 (*Hk1*), which harbored LFA in both Thevenard and Whitlock–Boullanger Islands; and desmoglein 3 (*Dsg3*), for which two LFA in close proximity (552 bp) were identified in both Thevenard and Whitlock–Boullanger Islands.

## 4 DISCUSSION

The application of homing endonuclease gene drives to management of rodent pest populations has attracted considerable attention (Campbell et al., 2019; Godwin et al., 2019), and recent laboratory studies have shown promising advancements in molecular techniques (Grunwald et al., 2019; Pfitzner et al., 2020). Successful deployment of such technologies will likely depend strongly on robust population genomic studies for taxonomic identification, selection of target populations, characterization of gene flow and invasion pathways, and development of safeguards to prevent unmitigated spread. However, with few exceptions (Schmidt et al., 2020) these types of studies have largely been neglected to date with respect to gene drive research. Here we performed a population genomic study of invasive mouse populations on four islands spanning a broad geographic range, with a particular focus on testing key assumptions of the LFA approach.

Our study provides several insights regarding the genetics of these populations as well as their suitability as (hypothetical) release sites for a gene drive biocontrol. Consistent with predictions of invasive populations on small and isolated oceanic islands, mice from island sites exhibited reduced genome‐wide allelic diversity. In the Honolulu population, which was established from anthropogenic introductions to the island of Oahu, both measures of allelic diversity (SNP‐*H*_e_ and Watterson's *θ*) were more similar to continental populations compared to other islands in the dataset (Figure 2a,b), suggesting a relatively large and genetically diverse population on Oahu and supporting its inclusion as a “continental” source population in our study design.

Evaluation of population genetic structure using PCA showed some degree of clustering between paired populations (e.g., Midway and Honolulu), likely due to historical demographic linkages or a direct island colonization event. At the same time, our estimates of *F*_ST_ imply moderate differentiation that is consistent with a reduction in gene flow, which is a critically important criterion for any candidate gene drive release site as ongoing migration could introduce resistance alleles to the target population. Analysis of population structure also revealed elevated allelic differentiation in the population from Broome, Western Australia, prompting an investigation into the potential inclusion of multiple *M*.* musculus* subspecies in our dataset. While previous studies have supported a strong predominance of *M*.* m*.* domesticus* in Australia (Gabriel et al., 2011), we found evidence of genomic admixture with *M*.* m* *castaneus* in this population, suggesting heretofore unappreciated introduction of this subspecies, most likely from its range in southeast Asia. This result highlights the importance of studies like ours that can provide taxonomic identification and reveal previously undetected immigration pathways that might impact island rodent eradication efforts.

Our results also provide several insights regarding the feasibility of the LFA approach for spatially limiting gene drive on islands. This strategy requires identification of Cas9 binding sites with genetic variants that are fixed within the target island population and preferably at a low allele frequency in nontarget populations. To counteract resistance alleles, LFA targets should be clustered closely together to facilitate multiplexed gRNA and occur within critical regions of haplosufficient female fertility genes that have minimal pleiotropic effects. Our results from analysis of four island–source population pairs suggest that, in practice, there may often be only a small number of sites that fit all of these criteria. The number of LFA targets identified was notably sensitive to the allele frequency cutoff in the “source” population. The absence of any suitable LFA when applying the strict filter (≤0.15) suggests that, for these islands, ongoing migration, insufficient time since colonization, stabilizing selection, or some combination of factors has prevented dramatic shifts in allele frequencies that are often observed on islands. At the other extreme, allowing for high allele frequencies (≤0.95) in the “source” population resulted in an expanded number of potential targets with multiple multiplex gRNA options. The trade‐off, however, is an increased risk of effects on nontarget populations in the event of a gene drive escape due to a higher frequency of susceptible alleles. While the gene drive is expected to rapidly be eliminated in such a scenario due to the presence of resistance alleles (Champer, Oakes, et al., 2020; Sudweeks et al., 2019), transient nontarget population impacts or even the public perception of increased risk of spread might prove unacceptable. Filtering LFA based on an intermediate “source” population allele frequency (≤0.50) resulted in a small number of sites, though several showed potential for multiplexed gRNA and occurred in genes of interest, and may therefore represent a reasonable compromise. Ultimately, the selection of a source population allele frequency cutoff would be a decision that managers make based on the assessed risk of escape from the island population and tolerance for any impacts on nontarget populations such as the rapid erosion of genetic diversity as resistant alleles replace susceptible alleles, or the unintended ecological release of competing pest species (e.g., *Rattus* spp.) into the niche vacated by suppressing mouse populations.

None of the LFA identified (at any allele frequency cutoff) were common across all populations. However, the absolute numbers of LFA identified were largely concordant across the four island–source pairs studied, which, with the exception of Broome and Thevenard Island, all showed similar levels of moderate population differentiation. The abundance of LFA is expected to increase with island population genetic differentiation, suggesting that the LFA approach may prove most relevant for extremely isolated and genetically differentiated islands. We also note that our analysis here considered only the canonical PAM for *S*.* pyogenes* Cas9, and the availability of alternative Cas9 variants (Hu et al., 2018) and other RNA‐guided endonucleases (Zetsche et al., 2015) afford a wide range of PAM compatibility that could permit fine tuning of LFA numbers and genomic locations.

Despite relatively low numbers of LFA targets overall, our analyses identified 40 genes that harbored LFA Cas9 targets. Further characterization highlighted three potential candidate genes with attractive properties for a population suppression gene drive application. All three candidate genes are associated with female infertility in homozygous knockouts, though evidence from the literature suggests that females lacking *Dsg3* are able to birth pups, but subsequently unable to maintain viable litters (Kountikov et al., 2015). Furthermore, there is evidence for haplosufficiency in each gene, with heterozygous individuals appearing fertile and grossly phenotypically normal, and with no apparent effects on male fertility (Kountikov et al., 2015; Peters et al., 2001; Wu et al., 2003). Disruption of *Hk1* leads to complete infertility in homozygous females, but also has broader deleterious effects on both sexes due to severe anemia (Peters et al., 2001) which may hinder efficient gene drive spread via males. Females lacking *Zar1* have normal ovary development and oogenesis, but embryos fail to develop past the single cell stage (Wu et al., 2003). Thus, the gene is hypothesized to mediate the oocyte‐to‐embryo transition and is therefore a particularly attractive target as an essential fertility gene with female‐specific expression and with potential for multiplexed gRNA. However, given that it is expressed in oocytes, its suitability as a gene drive target would first require experimentation to verify that homing does not disrupt oocyte viability in female carriers. More generally, we note that evidence for phenotypic effects for all of these genes comes from homozygous knockout experiments in mice from inbred laboratory lines, which may not always translate to wild‐type house mouse backgrounds. Thus, while the candidates identified through our study hold some promise, direct experimental validation is clearly necessary.

The reduced island allelic diversity observed across islands in our study suggests a strong role for genetic drift and founder effects in generating LFA. At the same time, we also detected a significant association between potential selective sweeps and LFA, with 38%–78% of LFA SNPs occurring within putative selective sweeps in each population. This is in itself perhaps unsurprising given that selective sweeps are characterized by regions of reduced diversity, but it does suggest a contributing role for selection in the presence of LFA in island mouse genomes. Selection could lead to LFA in island mouse genomes if positive selection for island‐selected phenotypes results in selective sweeps in associated regions. Our analysis of selective sweeps shared among island populations highlighted a gene set that was enriched for hormone function in general and growth hormone specifically. This result is interesting given that behaviors and body size are among the most commonly observed differences associated with insularity in rodent populations (i.e., “island syndrome,” Adler & Levins, 1994). Of particular note, introduced mice on Gough Island (Rowe‐Rowe & Crafford, 1992) and the Faroe Islands (Berry et al., 1978) have evolved dramatically increased body sizes, along with other behavioral and life history differences, after only a few hundred generations. Relevant to our results, developmental genetic studies of these other populations revealed accelerated growth in island mice during the first few weeks of life, when growth is largely regulated by the growth hormone‐IGF1 axis (Gray et al., 2015). Moreover, a genomic study contrasting island and continental mice found evidence of island‐specific selective sweeps surrounding loci controlling body size (Chan et al., 2012). While our study is strictly correlative and lacks the power to estimate the relative importance of selection, it suggests that introduced island mice might be subject to similar selective environments. Recurring evolution of island phenotypes may in turn provide common LFA that could be utilized across multiple islands, thereby avoiding the need to create a bespoke gene drive construct for each target island population. Thus, we propose that future investigations of island‐selected phenotypes could not only elucidate the value in targeting associated genomic regions for genetic biocontrol in these populations, but also provide insight into the genetic basis of the “island syndrome” by closer examination of the genes identified here.

In evaluating the overall feasibility of the LFA approach, there are several important considerations highlighted by our study. On the one hand, targeting LFA is attractive in part due to its relative technical simplicity, as it arguably would require no more sophisticated molecular components beyond the CRISPR‐Cas homing gene drive construct with a gRNA for each allele targeted. On the other hand, as with many other proposed gene drive strategies, the approach is sensitive to resistance alleles in the target population, as even low frequencies will dramatically undermine drive efficacy on islands (Unckless et al., 2017). Thus, successful application will depend critically on confidently identifying fixed allele targets as well as robust measures to curtail gene flow that might introduce resistant alleles to islands. The pooled sequencing approach utilized in this study provides a relatively cost‐effective technique for studying whole‐genome variation across multiple populations. However, in designing a pool‐seq assay, careful consideration should be paid to the risk of undetected resistance alleles segregating within island populations at very low frequencies (Figure S1). For example, under the sampling and sequencing scheme applied in this study, we can conservatively estimate a 44.7% probability that a locus with an actual minor allele frequency of 0.02 would be incorrectly identified as fixed (Figure S1, blue line). However, a doubling of both number of mice sampled and sequencing effort per pool is expected to reduce the probability of mis‐labeled LFA to <9% (Figure S1, red line), while tripling would further reduce the risk to <3% (Figure S1, green line). The incorporation of even more efficient genotyping techniques, such as custom hybrid‐capture sequencing assays that target LFA identified in an initial round of pool‐seq, could facilitate such high‐throughput genotyping at relatively low costs. Moreover, designs with multiplexed gRNA targeting LFA should further reduce this risk due to the lesser probability of selecting multiple loci that harbor rare alleles (Figure S1, dashed lines). We note that whereas the above calculations assume infinite population size in Hardy–Weinberg equilibrium, gene drives are likely to be most attractive for application in small isolated island populations where such rare alleles are expected to be uncommon due to genetic drift. In practice, it may be important to develop island‐specific population models to inform design of genetic assays that balance efficiency with the estimated risk of undetected rare alleles.

In conclusion, we note that, while only a small fraction of sites fit the criteria for LFA given the gene drive design considered, our scans of genomic variation in island mice identified thousands of SNPs within Cas9 target sites in genic CDS and 5’UTR that were fixed in the island populations. This result is similar to a recent study of wild mosquito populations that found abundant fixed Cas9 targets in protein‐coding regions (Schmidt et al., 2020), and therefore provides a promising first population genetic assessment of gene drive potential for control of invasive mice on islands, as targeting such regions implies a lesser chance of resistance alleles already segregating in the population. The added benefit gained by targeting LFA can be viewed as one of numerous proposed safeguards, and it is possible that future applications may employ redundant combinations of molecular and field‐based biocontainment mechanisms such as the targeted use of rodenticides to augment genetic biocontrol releases. Overall, it is becoming clear that, regardless of approach, thorough population genomic surveys of target populations in the field will be key for both informed gene drive design (Schmidt et al., 2020) and understanding population structure and evolutionary history. Ultimately, the feasibility of any gene drive design for control of invasive rodents on islands will depend on a multidisciplinary assessment of risks and benefits with respect to biological, economic, social, and ethical factors (Campbell et al., 2015; Godwin et al., 2019; Hayes et al., 2018; Taitingfong, 2019).

## Supporting information

Supplementary Material

Table S4

Table S6

## Data Availability

Aligned whole‐genome sequence data from this study are available at the NCBI Sequence Read Archive under BioProject accession PRJNA702596.
