# Supplementary material for: Population genomics of invasive rodents on islands: Genetic consequences of colonization and prospects for localized synthetic gene drive
Source: Evol Appl. 2021 Mar 10;14(5):1421–35. doi: 10.1111/eva.13210 (PMC8127709; doi:10.1111/eva.13210)
Supplement: Supplementary file 1 — Supplementary Material [file EVA-14-1421-s002.docx]

**Table S1.** Mouse tissue sampling summary. Paired island (grey shaded) and source populations (unshaded) are grouped within solid lines, with approximate straight line (Euclidean) distances between populations provided in fourth column.

| Population | N | Tissue type | Approx. distance (km) |
| --- | --- | --- | --- |
| Sand Island, Midway Atoll | 40 | tail snips | 2,102 |
| Honolulu Airport, Oahu, Hawai’i | 40 | tail snips |  |
| Southeast Farallon Island, California | 40 | tail snips | 55 |
| San Francisco Bay, California | 23 | tail snips |  |
| Whitlock-Boullanger Islands, W. Australia | 40 | ear punch | 4 |
| Jurien Bay, W. Australia | 40 | ear punch |  |
| Thevenard Island, W. Australia | 40 | ear punch | 850 |
| Broome, W. Australia | 40 | ear punch |  |

**Table S2.** Mouse samples provided under tissue grant from the Museum of Vertebrate Zoology, University of California, Berkeley.

| **Sample ID** | **Sample Source** |
| --- | --- |
| MVZ:Mamm:224834 | CA, USA |
| MVZ:Mamm:220304 | CA, USA |
| MVZ:Mamm:218009 | CA, USA |
| MVZ:Mamm:206285 | CA, USA |
| MVZ:Mamm:218002 | CA, USA |
| MVZ:Mamm:220303 | CA, USA |
| MVZ:Mamm:216737 | CA, USA |
| MVZ:Mamm:216748 | CA, USA |
| MVZ:Mamm:216735 | CA, USA |
| MVZ:Mamm:216743 | CA, USA |
| MVZ:Mamm:216736 | CA, USA |
| MVZ:Mamm:216744 | CA, USA |
| MVZ:Mamm:216740 | CA, USA |
| MVZ:Mamm:216734 | CA, USA |

**Table S3.** Pooled whole-genome resequencing (150 bp paired-end) and mapping (to GRCm38 reference genome) statistics.

| Population | Yield (Gbp) | Mean read quality (Phred) | Mean coverage | % reference bases covered | Mean mapping quality (Phred) |
| --- | --- | --- | --- | --- | --- |
| Sand Island, Midway Atoll | 390.7 | 37.2 | 90.0X | 96.3 | 49.0 |
| Honolulu Airport, Oahu, Hawai’i | 268.0 | 32.2 | 82.5X | 96.5 | 47.8 |
| South Farallon Island, California | 137.1 | 32.8 | 42.4X | 96.1 | 48.6 |
| San Francisco Bay, California | 143.0 | 32.7 | 46.8X | 96.3 | 47.2 |
| Whitlock-Boullanger Isls, W. Australia | 135.0 | 32.1 | 41.5X | 95.9 | 48.1 |
| Jurien Bay, W. Australia | 124.9 | 33.3 | 42.1X | 96.1 | 48.5 |
| Thevenard Island, W. Australia | 118.3 | 33.1 | 40.0X | 96.1 | 48.9 |
| Broome, W. Australia | 156.7 | 32.8 | 52.2X | 96.2 | 48.2 |

**Table S5.** Enriched functional terms from GO analysis of genes associated with selective sweeps in island mouse populations. Both unadjusted (modified Fisher exact score) and adjusted *P-*values (Benjamini-Hochberg adjusted) are provided.

| **Functional Term** | **Gene Count** | **Fold Enrichment** | **P-Value** | **P-Value (adjusted)** |
| --- | --- | --- | --- | --- |
| Somatotropin hormone | 16 | 20.87 | 7.8e-17 | 1.1e-13 |
| Phosphoprotein | 283 | 1.39 | 1.5e-11 | 4.8e-09 |
| Cytoplasm | 185 | 1.57 | 4.4e-11 | 7.0e-09 |
| Hormone | 20 | 6.92 | 7.0e-11 | 7.4e-09 |
| Somatotropin hormone, conserved site | 11 | 21.14 | 1.6e-11 | 8.0e-09 |
| cytoplasm | 255 | 1.37 | 4.6e-10 | 2.0e-07 |
| Four-helical cytokine, core | 17 | 7.05 | 1.9e-09 | 6.2e-07 |
| Four-helical cytokine-like, core | 17 | 6.82 | 3.1e-09 | 7.7e-07 |
| Alternative splicing | 185 | 1.45 | 3.3e-08 | 2.6e-06 |
| hormone activity | 15 | 4.76 | 3.1e-06 | 2.1e-03 |
| Nucleus | 161 | 1.33 | 6.6e-05 | 4.2e-03 |
| Autophagy | 13 | 3.95 | 1.2e-04 | 6.0e-03 |
| ATP-binding | 60 | 1.64 | 1.8e-04 | 8.1e-03 |
| enzyme binding | 27 | 2.50 | 3.4e-05 | 1.2e-02 |
| neuronal cell body | 32 | 2.14 | 1.1e-04 | 2.3e-02 |
| Nucleotide-binding | 70 | 1.49 | 7.9e-04 | 2.7e-02 |
| Kinase | 35 | 1.85 | 7.6e-04 | 3.0e-02 |
| Ubl conjugation | 61 | 1.51 | 1.3e-03 | 3.9e-02 |

**Table S7.** Candidate female mouse fertility genes for that harbor locally-fixed alleles (LFA, allele frequency in ‘source’ population $\boldsymbol{\leq}$ 0.50). Counts indicate the number of LFA Cas9 targets for each island population occurring in the gene CDS or 5’UTR. The number of potential multiplex sets identified (i.e., two or more LFA SNPs occurring within a 500bp window) are provided in parentheses. Last column indicates knockout phenotypes of each gene that may impact suitability of gene in a population suppressing gene drive application: (a) male infertility/reduced fertility, (b) abnormal gametogenesis, (c) abnormal oogenesis, (d) abnormal meiosis.

|  |  |  | No. LFA observed (No. multiplex sets) | | | |  |  |
| --- | --- | --- | --- | --- | --- | --- | --- | --- |
| Ensembl ID | Symbol | Name | Farallon | Midway | Thevenard | Whitlock-Boullanger | | Notes |
| ENSMUSG00000026039 | *Sgo2a* | shugoshin 2A | 4 (2) | – | – | 1 | | a,b,d |
| ENSMUSG00000019971 | *Cep290* | centrosomal protein 290 | 1 | – | – | – | | a |
| ENSMUSG00000021820 | *Camk2g* | ca/calmodulin-dependent protein kinase II gamma | 1 | – | – | – | |  |
| ENSMUSG00000002324 | *Rec8* | REC8 meiotic recombination protein | 2 | – | – | – | | a,b,c,d |
| ENSMUSG00000022096 | *Hr* | lysine demethylase and nuclear receptor corepressor | 1 | – | – | – | |  |
| ENSMUSG00000005268 | *Prlr* | prolactin receptor | 1 | – | – | – | | a,c |
| ENSMUSG00000016763 | *Scube1* | signal peptide, CUB domain, EGF-like 1 | 2 (1) | – | – | – | | a |
| ENSMUSG00000022479 | *Vdr* | vitamin D (1,25-dihydroxyvitamin D3) receptor | 1 | – | – | – | | a,b |
| ENSMUSG00000023830 | *Igf2r* | insulin-like growth factor 2 receptor | 1 | – | – | – | | a |
| ENSMUSG00000024429 | *Gnl1* | guanine nucleotide binding protein-like 1 | 1 | – | – | – | | a |
| ENSMUSG00000052105 | *Mtcl1* | microtubule crosslinking factor 1 | 1 | – | – | – | |  |
| ENSMUSG00000024413 | *Npc1* | NPC intracellular cholesterol transporter 1 | 2 | – | – | – | | a |
| ENSMUSG00000026976 | *Pax8* | paired box 8 | 1 | – | – | – | | a |
| ENSMUSG00000027832 | *Ptx3* | pentraxin related gene | 1 | – | – | – | |  |
| ENSMUSG00000043410 | *Hfm1* | HFM1, ATP-dependent DNA helicase homolog | 1 | – | – | 2 | | a,b,c,d |
| ENSMUSG00000031090 | *Nadsyn1* | NAD synthetase 1 | 1 | – | – | – | |  |
| ENSMUSG00000020593 | *Lpin1* | lipin 1 | – | 1 | – | – | | a |
| ENSMUSG00000036678 | *Aaas* | achalasia, adrenocortical insufficiency, alacrimia | – | 1 | – | – | |  |
| ENSMUSG00000054932 | *Afp* | alpha fetoprotein | – | 1 | – | – | |  |
| ENSMUSG00000041147 | *Brca2* | breast cancer 2, early onset | – | 3 | – | – | | a |
| ENSMUSG00000029736 | *Nobox* | NOBOX oogenesis homeobox | – | 1 | – | – | | c |
| ENSMUSG00000030451 | *Herc2* | HECT and RLD domain containing E3 ubiquitin protein ligase 2 | – | 1 | – | – | | a |
| ENSMUSG00000037012 | *Hk1* | hexokinase 1 | – | – | 1 | 1 | |  |
| ENSMUSG00000052593 | *Adam17* | a disintegrin and metallopeptidase domain 17 | – | – | 1 | – | | a |
| ENSMUSG00000056632 | *Dsg3* | desmoglein 3 | – | – | 2 | 2 | |  |
| ENSMUSG00000029026 | *Trp73* | transformation related protein 73 | – | – | 1 | – | | a,d |
| ENSMUSG00000044674 | *Fzd1* | frizzled class receptor 1 | – | – | 1 | 1 | |  |
| ENSMUSG00000063935 | *Zar1* | zygote arrest 1 | – | – | 2 (1) | – | |  |
| ENSMUSG00000029338 | *Antxr2* | anthrax toxin receptor 2 | – | – | 1 | – | |  |
| ENSMUSG00000039842 | *Mcph1* | microcephaly, primary autosomal recessive 1 | – | – | 1 | – | | a,d |
| ENSMUSG00000020063 | *Sirt1* | sirtuin 1 | – | – | – | 1 | | a,d |
| ENSMUSG00000040711 | *Sh3pxd2b* | SH3 and PX domains 2B | – | – | – | 1 | | a |
| ENSMUSG00000022432 | *Smc1b* | structural maintenance of chromosomes 1B | – | – | – | 1 | | a,d |
| ENSMUSG00000022914 | *Brwd1* | bromodomain and WD repeat domain containing 1 | – | – | – | 1 | | a,d |
| ENSMUSG00000058152 | *Chsy3* | chondroitin sulfate synthase 3 | – | – | – | 2 (1) | | a |
| ENSMUSG00000073889 | *Il11ra1* | interleukin 11 receptor, alpha chain 1 | – | – | – | 1 | |  |
| ENSMUSG00000028469 | *Npr2* | natriuretic peptide receptor 2 | – | – | – | 1 | | a,c,d |
| ENSMUSG00000057722 | *Lepr* | leptin receptor | – | – | – | 1 | | a |
| ENSMUSG00000000440 | *Pparg* | peroxisome proliferator activated receptor gamma | – | – | – | 1 | |  |
| ENSMUSG00000040734 | *Ppp1r13l* | protein phosphatase 1, regulatory subunit 13 like | – | – | – | 1 | | a |

**Figure S1.** Effect of rare allele frequency on the probability of incorrectly labeled fixed allele in island mice. For the purpose of these calculations, we used the same pool-seq sampling design (N=40 mice per population) and mean sequencing coverage per library (~55X) observed in our study (blue lines), as well as scenarios with twice (red lines) and three times (green lines) the sampling and sequencing efforts per pool. For each scenario, we consider both single gRNA (solid lines) and multiplex (2 gRNA) designs (dashed lines). All calculations assume populations of infinite size in Hardy-Weinberg equilibrium, with zero variance in individual representation within pools, and uniform sequencing coverage across loci.

The probability of mis-labeling LFA (*Q*) for designs with a single gRNA was calculated as a function of true minor allele frequency (*p*) as follows:

$$Q\left( p \right)=\sum_{i =0}^{2N} \left[ P\left( i;2N,p \right) \times P\left( 0;C,\frac{i}{2N} \right) \right]$$

Here, $P\left( i;2N,p \right)$ represents the probability of drawing value *i* from a binomial distribution with parameters *N* and *p*. Hence the first term represents the probability of *i* minor alleles occurring among the 2*N* chromosomes sampled from the population. The second term, $P\left( 0;C,\frac{i}{2N} \right)$, represents the probability of sequencing zero minor alleles among the *C* sequencing reads from each pooled sequencing library. For estimates with two gRNA, an additional term for the second locus was included:

$$Q\left( p \right)=\sum_{i =0}^{2N} \left[ P\left( i;2N,p \right) \times P\left( 0;C,\frac{i}{2N} \right)\times P\left( 0;C,\frac{i}{2N} \right) \right]$$
